# Multimodal imaging analyses of hyperreflective dot-like lesions in acute syphilitic posterior placoid chorioretinopathy

**DOI:** 10.1186/s12348-016-0119-7

**Published:** 2017-01-12

**Authors:** Luiz H. Lima, Gabriel Costa de Andrade, Silvana Vianello, Claudio Zett, Michel E. Farah, Rubens Belfort

**Affiliations:** 1 Department of Ophthalmology, Federal University of São Paulo, São Paulo, Brazil; 2 Federal University of São Paulo (UNIFESP), Rua Botucatu, 821, Vila Clementino, São Paulo, SP 04023-062 Brazil; 3 Pontificia Universidad Católica de Valparaíso, Valparaíso, Chile

**Keywords:** Acute syphilitic posterior placoid chorioretinitis, En-face optical coherence tomography, Spectral-domain optical coherence tomography, Syphilis, Outer retinal abnormalities

## Abstract

**Background:**

Retrospective review of one acute syphilitic posterior placoid chorioretinitis (ASPPC) case with serological evidence of syphilis who had ocular signs and symptoms not attributable to other diseases. Enface and spectral-domain optical coherence tomographySD-OCT were analyzed at the time of presentation and at 1-month visit following initiation of treatment. The study patient underwent standard treatment for neurosyphilis.

**Results:**

Ophthalmic examination and imaging studies were consistent with the diagnosis of ASPPC. The patient age was 33 year-old and the baseline visual acuity was 20/400 and 20/80 in the right and left eyes, respectively. At presentation, SD-OCT scans showed disruption and loss of the ellipsoid zone (EZ), small nodular elevations on retinal pigment epithelium (RPE) and punctate hyperreflectivity in the choroid. Enface OCT at the level of RPE and EZ demonstrated multiple hyperreflective dot-like lesions simmetrically distributed within the macular area. These dot-like lesions corresponded to the small nodular elevations on RPE and to disruption/loss of EZ observed with SD-OCT. One month after neurosyphilis therapy, the visual acuity improved and the outer retinal changes partially reversed in both eyes.

**Conclusions:**

We report the outer retinal findings and its correlation using both en-face and SD-OCT in a patient with ASPPC. En-face OCT imaging provides a more precise outer retinal layers analyses allowing a better understanding of the ASPPC pathophysiology.

## Findings

Acquired syphilis is a chronic spirochete infection caused by the *Treponema pallidum* and may cause serious systemic lesions. Although the new cases of syphilis decreased greatly in the second half of the 20th century, the incidence of syphilis increased among men in recent years, accounting for about 67% of syphilis cases in the USA. The World Health Organization estimated that the worldwide annual incidence of syphilis is approximately 11 million in adults with the majority of these cases (about 90%) occurring in developing countries [1–4].

Eye involvement occurs in 5 to 8% of syphilis cases, has been reported at all disease stages, and develops more frequently during the secondary and tertiary stages of the disease. Syphilis is usually named as the great masquerader of ocular diseases due to the broad spectrum of signs. Ocular manifestations include uveitis, interstitial keratitis, iritis, vitritis, chorioretinitis, serous retinal detachment, and papillitis. Posterior uveitis is the most frequent complication, and chorioretinitis is the most common posterior segment manifestation [5–7].

Acute syphilitic posterior placoid chorioretinitis (ASPPC) is an uncommon expression of syphilis and was reported to describe a round, large, yellowish, placoid lesion at the level of the retinal pigment epithelium (RPE) in the macular area or posterior pole (within the arcades) [8–10]. Fluorescein (FA) and indocyanine green (ICG) angiography usually demonstrate early central hypo or hyperfluorescence followed by late hyperfluorescence (staining) [11, 12]. On spectral-domain optical coherence tomography (SD-OCT), ASPPC may lead to outer retinal changes such as disruption and loss of both the ellipsoid zone (EZ) and external limiting membrane (ELM), nodular thickening of the RPE, and accumulation of subretinal fluid [13, 14]. In this case report, we correlate both the en-face and SD-OCT findings of the outer retina along with the clinical evolution of one patient diagnosed with ASPPC.

### Case report

**Fig. 1:**
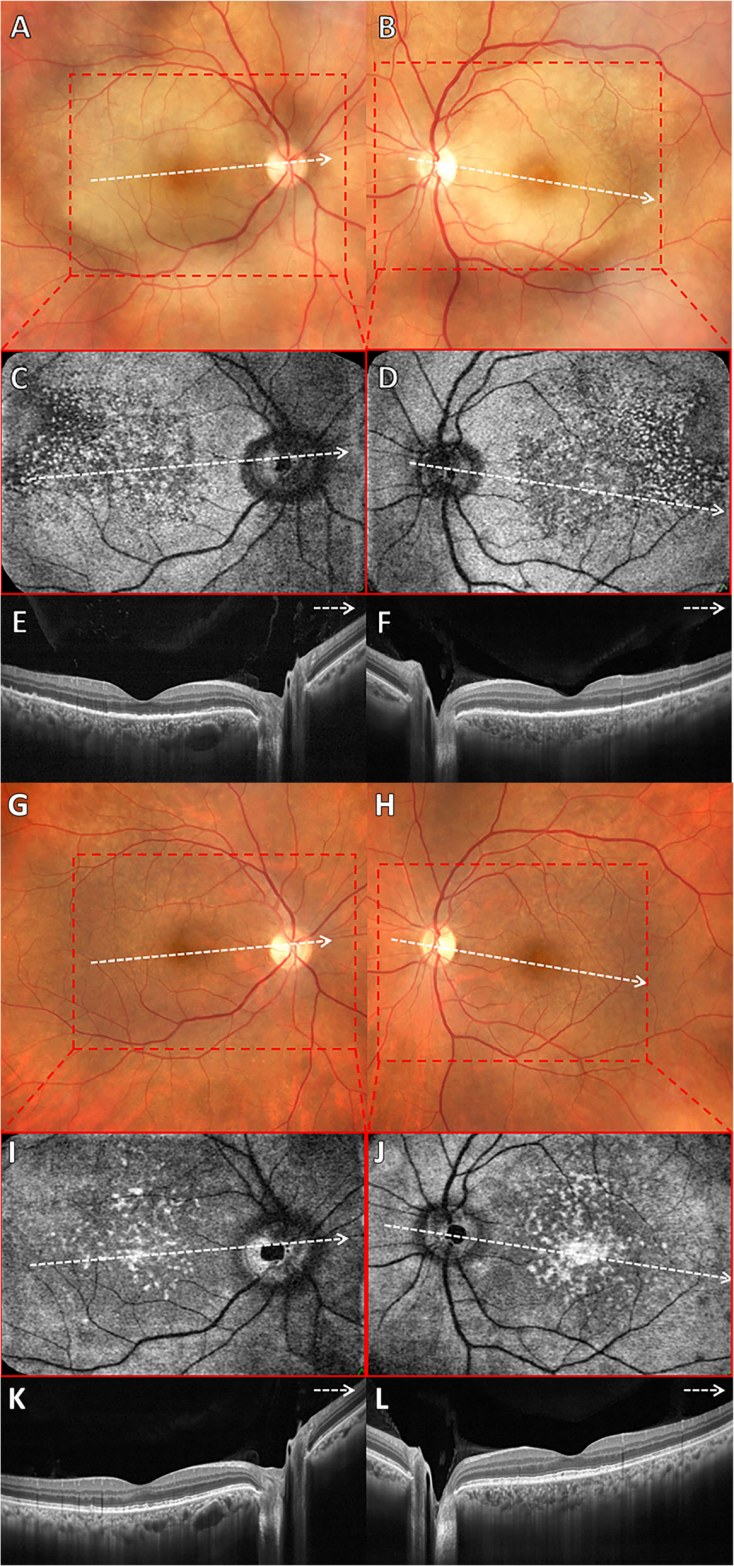
**a**, **b** Color fundus photograph of both eyes revealed a yellowish and placoid lesion within the posterior pole. **c**, **d** En-face OCT at the level of EZ and RPE demonstrated multiple hyperreflective dots uniformly distributed within the macular area of both eyes corresponding to the RPE nodular elevations observed with SD-OCT. **e**, **f** B-scan of spectral-domain optical coherence tomography (SD-OCT) of both eyes revealed an intact external limiting membrane (ELM), disruption and loss of the ellipsoid zone (EZ), small nodular elevations on retinal pigment epithelium (RPE) and punctate hyperreflectivity in the choroid. **g**, **h** At 1-month follow-up, color fundus photograph showed disappearance of placoid lesion in both eyes. **i**, **j** At 1-month follow-up, en-face OCT at the level of EZ and RPE showed partial disappearance of hyperreflective dots within the macular area of both eyes. **k**, **l** At 1-month follow-up, B-scan of SD-OCT of both eyes demonstrated remodeling of the EZ band and nearly total resolution of RPE nodular elevations and punctate hyperreflectivity in the choroid

**Fig. 2:**
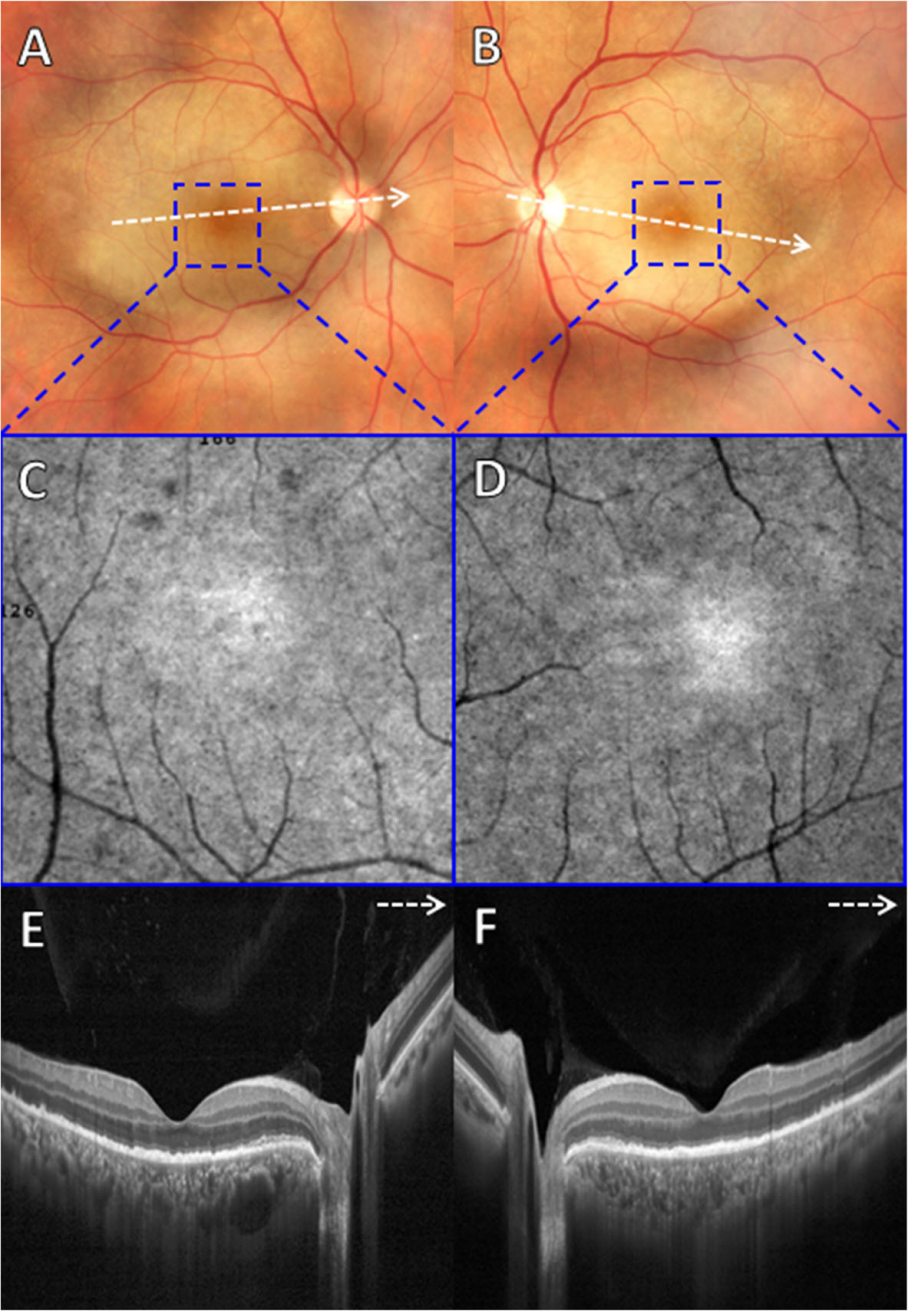
**a**, **b** Color fundus photograph of both eyes revealed a yellowish and placoid lesion within the posterior pole. **c**, **d** En-face OCT angiography 3 × 3 mm at the level of outer retina demonstrated multiple hyperreflective dots uniformly distributed within the foveal area of both eyes. **e**, **f** B-scan SD-OCT of both eyes revealed an intact ELM, disruption and loss of EZ, small nodular elevations on RPE and punctate hyperreflectivity in the choroid. **g**, **h** At 1-month follow-up, color fundus photograph showed disappearance of placoid lesion in both eyes. **i**, **j** At 1-month follow-up, en-face OCT angiography 3 × 3 mm at the level of outer retina showed partial disappearance of hyperreflective dots within the foveal area of both eyes. **k**, **l** At 1-month follow-up, B-scan SD-OCT of both eyes demonstrated remodeling of the EZ band and nearly total resolution of RPE nodular elevations and punctate hyperreflectivity in the choroid

A 33-year-old woman presented with sudden decrease of visual acuity in both eyes. The patient had a previous history of maculopapular rash on the trunk in conjunction with fever and headache 1 month before the visual loss. On ocular examination, best-corrected visual acuity (BCVA) was 20/400 in the right eye and 20/80 in the left eye. Biomicroscopy of anterior segment revealed cells 1+ in the anterior chamber in both eyes. Pupillary reactions and intraocular pressure were normal, and vitreous cells 3+ were observed in both eyes. Color fundus photograph of both eyes revealed a yellowish placoid lesion in the posterior pole, including the macula. FA depicted punctate central hypofluorescence followed by progressive hyperfluorescence in the area of the lesion in both eyes. The B-scan OCT (Optovue RTVue 100, Optovue Inc, Fremont, CA, USA) of both eyes revealed an intact ELM, disruption and loss of EZ, small nodular elevations on the RPE, and punctate hyperreflectivity in the choroid. En-face OCT at the level of RPE and EZ demonstrated multiple hyperreflective dot-like lesions symmetrically distributed in the macular area of both eyes. These dot-like lesions corresponded to the small nodular elevations on RPE observed with B-scan OCT (Fig. 1). Laboratory tests revealed reactivity to both the venereal disease research laboratory (VDRL) (1/512) and fluorescent treponemal antibody absorption (FTA-ABS) tests. Serum anti-HIV antibodies search was negative. The patient underwent the standard treatment for neurosyphilis (intravenous penicillin G at a dosage of 24 million units per day for 14 days). After 1-month follow-up, there were partial reformation of the EZ band and nearly total resolution of nodular elevations on RPE and punctate hyperreflectivity in the choroid. En-face OCT at the level of EZ and RPE demonstrated partial disappearance of hyperreflective dots within the macular area of both eyes (Fig. 1). BCVA improved to 20/60 in the right eye and 20/40 in the left eye. The en-face OCT angiography 3 × 3 mm at the level of outer retina demonstrated multiple hyperreflective dots uniformly distributed within the foveal area of both eyes (Fig. 2a–f) that partially disappeared at 1-month follow-up (Fig. 2g–l).

### Discussion

ASPPC is referred as a variant of syphilis chorioretinitis represented by the presence of a unique or several yellowish and placoid-like lesions within the posterior pole [8–10]. Our case showed similar retinal findings of other reports of ASPPC in the literature depicting large, circular, placoid lesions located within the posterior pole. The characteristic ASPPC FA features of early central hypofluorescence and late hyperfluorescent staining were also observed in this case. It is recommended that the screening for syphilis should include both the FTA-ABS and VDRL testing since FTA-ABS may lead to false negative results and VDRL can give information about the activity of the disease [13]. The patient tested positive for both VDRL and FTA-ABS and tested negative for HIV. Although some reports have suggested an association between the occurrence of ASPPC and HIV infection, the clinical aspects of ASPPC usually do not differ when HIV-positive patients are compared with HIV-negative patients [12, 15, 16].

In the present case, SD-OCT showed common findings of hyperreflective nodularity of the RPE, loss and disruption of EZ band, and punctate hyperreflectivity in the choroid. Similar SD-OCT outer retinal changes were previously described. Brito et al. [14] reported an acute disruption of the outer retinal bands, irregular hyperreflectivity with nodular elevation of the RPE, and punctate hyperreflectivity in the choroid. Pichi et al. [13] also observed an irregular hyperreflectivity with nodular elevations at the junction of the photoreceptors and the RPE associated with segmental loss of the EZ. In addition, hyperreflective pinpoint lesions in the choroid have been previously described in the literature [13]. Although subretinal fluid (SRF) and external ELM disruption have been observed on OCT scans of syphilis patients [12–14, 17], our case did not show similar changes. As SRF is a very early feature of APPC and its incidence in ASPPC varies between 11.8 and 43.3% [12, 13], the lack of SRF in our case may be due to the later performance of SD-OCT. En-face scans at the level of RPE and EZ demonstrated several roundish hyperreflective lesions that appeared confluent in the parafoveal area and corresponded to the hyperreflective nodularity of the RPE seen on SD-OCT. The en-face images at the level of the choriocapillaris also depicted hyperreflective pinpoint lesions in the study eyes.

Initiation of systemic penicillin therapy led to prompt normalization of vision and restoration of outer retinal and choroidal anatomy. At 1-month follow-up, SD-OCT following antibiotic treatment demonstrated partial disappearance of the pathologic changes observed within the macula, with reformation of the EZ band, the RPE, and the choroid. En-face OCT at the level of RPE and EZ also showed incomplete resolution of the hyperreflective dot-like lesions in the macular area after 1 month of treatment initiation. This anatomical restoration was related to visual acuity improvement that varied from 20/20 to 20/60 (mean, 20/30) at the last follow-up (1 month). The disappearance of these outer retinal features and nearly complete normalization of visual acuity following the standard syphilis therapy has been described in other previous reports [13, 14]. In our case, there would be a complete disappearance of the outer retinal lesions and a total visual acuity return to normal levels whether the follow-up was longer than 1 month.

Although the pathophysiology of ASPPC is not completely understood, the first two reports of this condition postulated that an inflammatory reaction or immune complex deposition at the level of the choroid-RPE-photoreceptor complex would lead to the clinical appearance of the placoid lesion and photoreceptor dysfunction [9, 10]. Previous studies using indocyanine green angiography (ICGA), fundus autofluorescence (FAF), and SD-OCT have suggested that the ASPPC lesions are located at the choroid or RPE. ICGA of such lesions demonstrates hypofluorescence probably due to choriocapillaris inflammation or retention of degraded material from the RPE and photoreceptor outer segments. FAF shows hyperautofluorescence corresponding to the lesions topography that is compatible with the accretion of lipofuscin or photoreceptor outer segments in the RPE [11–14, 18–20]. In our patient, the en-face OCT and SD-OCT findings apparently suggested a pathogenic process at the level of the choroid and RPE that had consequences in the photoreceptors (EZ layer) and were reversible with proper antibiotic treatment. Nonetheless, the pathogenesis of ASPPC is still unknown; we may postulate the sequence of disease events based on the correlation of our en-face and SD-OCT findings. Possibly, the choroidal hyperreflective pinpoint lesions seen on SD-OCT are consistent with inflammatory foci in the choroid vasculature as the circulating *T. pallidum* organisms enter the outer retina through the choroidal circulation [10]. One may speculate that the presence of treponemes in the choroid could result in the development of antibodies which, successively, may cause transient focal choroidal thrombosis and RPE structure disorganization represented by the hyperreflective nodularity (SD-OCT) and hyperreflective dot lesions (en-face OCT) on RPE, resulting in impaired photoreceptor function expressed by disruption/loss (SD-OCT) and hyperreflective dots (en-face OCT) on EZ.

To the best of our knowledge, this is the first ASPPC patient analyzed with en-face OCT. Recently, using en-face OCT, Sridhar et al. [21] published the disappearance of outer retinal dots in only 1 patient treated for syphilitic chorioretinitis. Their findings were consistent with ours (i.e., hyperreflective dots at the outer retina), but they did not specify which outer retinal layers were involved. In conclusion, en-face OCT imaging may allow the assessment of ASPPC with a new level of anatomic detail and may determine better pathophysiology knowledge of this particular type of syphilitic chorioretinitis.
